# A “Green” Stirring Plasma Functionalization Strategy for Controllable Oxygen-Containing Functional Groups on Octa-Methyl POSS Microstructure

**DOI:** 10.3390/nano13202770

**Published:** 2023-10-16

**Authors:** Xiao Chen, Kevin Magniez, Pengchao Zhang, Wojciech Kujawski, Zhiqiang Chen, Ludovic F. Dumée

**Affiliations:** 1 State Key Laboratory of Advanced Technology for Materials Synthesis and Processing, School of Materials Science and Engineering, Wuhan University of Technology, 122 Luoshi Road, Wuhan 430070, China; xiao.chen9013@gmail.com (X.C.); pczhang@whut.edu.cn (P.Z.); 2 Sanya Science and Education Innovation Park, Wuhan University of Technology, Sanya 572024, China; 3 Institute for Frontier Materials, Deakin University, 75 Pigdons Road, Waurn Ponds, Geelong, VIC 3216, Australia; kevinmagniez@textortechnologies.com; 4 Faculty of Chemistry, Nicolaus Copernicus University in Toruń, 7 Gagarina Street, 87-100 Toruń, Poland; wkujawski@umk.pl; 5 Textor Technologies PTY LTD, 41 Tullamarine Park Road, Tullamarine, VIC 3043, Australia; 6 Khalifa University, Department of Chemical Engineering, Abu Dhabi, United Arab Emirates; 7 Research and Innovation Center on 2D Nanomaterials, Arzanah Precinct, Abu Dhabi, United Arab Emirates

**Keywords:** plasma treatment, selective grafting, functionalization, POSS, carboxyl groups, hydroxyl groups

## Abstract

The distinctive cage-like structure of polyhedral oligomeric silsesquioxane (POSS) materials makes them highly effective fillers in composite membranes for separation applications. However, realizing their full potential in the application often requires specific surface functionalization with various groups. However, this requirement remains challenging owing to the limitations of wet-chemistry approaches, which frequently result in the generation of hazardous chemical by-products. In this paper, a “green” stirring plasma strategy is presented for the functionalization of octa-methyl POSS sub-micron particles into designable oxygen-containing functional groups using a low-pressure oxygen plasma from combined continuous wave and pulsed (CW+P) modes. Plasma from oxygen gas with CW mode offers highly oxygen-reactive species to continuously etch and activate the surface of the POSS. The resulting pulsed plasma assists in grafting more reactive oxygen species onto the active methyl groups of the POSS to form specific oxygen-containing functional groups including hydroxyl and carboxyl. A precise control of nearly one hydroxyl or one carboxyl group at the corner of the cage structure of the POSS is demonstrated, without damaging the core. Therefore, the plasma process discussed in this work is suggested by the authors as controllable fundamental research for the surface functionalization of sub-micron particles, promoting a more environmentally friendly pathway for the preparation of designable fillers.

## 1. Introduction

The interaction between fillers and a matrix is highly affected by their chemical and physical natures, which ultimately shape the performance of a composite in membrane separation and biomaterials [1,2]. Therefore, fillers with specific functionalities and dimensions are important for designing nanocomposites with desired properties [3,4,5,6,7]. Fillers such as silica, graphene, MXene, and metal organic frameworks (MOFs) have been shown to provide selective routes for enhancing the permeation of selected components from composite membranes [8,9,10,11]. Compared to other organic or inorganic fillers, polyhedral oligomeric silsesquioxanes (POSS) provide an inorganic core with organic functional groups with a size of 1–3 nm as a distinctive chemical structure which shows possibilities in membranes [12].

Specifically, POSS are inorganic silica-cage nanobuilding blocks containing a variety of functional groups in their corner sites. Such versatility in functionality has enabled applications in a wide range of composites [13,14]. In membrane-based materials, the use of POSS has received increased attention owing to demonstrated enhancements in anti-fouling and permeation [15,16,17,18]. By leveraging diverse functionalities, POSS can be used for different membrane applications. For example, the octa-methyl POSS was used for solvent separation membranes with a high ethanol permeation [19], while an octa-ammonium POSS was used for reverse osmosis (RO) membranes to enhance the water flux without losing salt rejection [20].

However, the development of functional POSS microstructures containing specific functionality relies on synthetic chemical wet methods. They are usually based on multi-stage modifications involving hydrolysis of silica derivatives and condensation processes of the native POSS precursor [12,13]. The array of reactive agents, solvents, and potential sub-products limits this method’s ability to become simple and cost-effective. Hence, the development of alternative functionalization methods for POSS materials that are efficient, tailorable, and environmentally friendly is still gaining attention [12,13,21]. Physical solutions such as thermal and plasma treatments are being proven to result in much less environmental waste compared to wet-chemical routes [22,23,24].

The plasma process provides reactive species from gases or gaseous materials, including ions, electrons, radicals, and radiations [25,26]. Controllable species are generated from oxygen plasma on the surface of materials, which mainly provide reactive radicals (O^•^, O_2_^•^) for forming oxygen-containing functional groups [27,28]. It has been reported that the etching of organic components and the formation of functional groups occur simultaneously from oxygen plasma [29]. Hence, studies on low-pressure oxygen plasma functionalization of polystyrene (PS), graphene oxides (GO), and multi-wall carbon nanotubes (MWCNTs) have indicated the successful introduction of hydroxyl, carbonyl, and carboxyl groups [28,30,31,32,33]. In addition, the substitution of H atoms by OH was proven on the polymeric part of the material, which accelerated the surface oxidation [34].

Nevertheless, plasma treatment has limited ability to achieve uniform modification of particles in micron- to nanoscale owing to their aggregation [35,36,37]. Thus, it has been reported that the homogeneity of plasma on such materials can be improved by using a number of techniques [37,38,39]. For instance, a bespoke stirring system for plasma was reported to have resulted in effective polymerization of heptylamine (HA) on MWCNTs for reinforcing the epoxy nanocomposite [37]. The stirring process allowed for a continuous exposure of the plasma glow to small-size particles and offered an increased contact area for modification. Moreover, the combination of continuous wave (CW) and pulsed (P) plasma modes indicated more precise control for introducing various functional groups onto the nanomaterials [35,36,37].

In this study, a bespoke stirring plasma system was used to treat octa-methyl POSS sub-micron particles using an oxygen gas plasma. The dissociated methyl groups from POSS cages during plasma were subsequently reacted with active oxygen species, inducing the formation of oxygen-rich functional groups [30]. Different energy inputs were demonstrated to investigate the impact of plasma on the grafting of hydroxyl and carboxyl groups. The bond dissociation energy is important in the oxygen plasma functionalization so that the applied kinetic energy can exactly activate C-H (3.5 eV) while maintaining the Si-C (4.5 eV) and the cage-structural Si-O (8.3 eV) [28,34,40]. The effectiveness and applicability of this novel method will be explored as the fundamental research in order to establish a green functionalization strategy.

## 2. Materials and Methods

### 2.1. Materials

The octa-methyl POSS (>97%) for plasma functionalization were commercially provided by Hybrid Plastics Inc.,Hattiesburg, MS, USA [24]. The POSS particles that were supplied presented as agglomerates and exhibited a large particle size (25 to 100 µm).

### 2.2. Sample Preparation

Owing to the large size of the supplied POSS, the plasma treatment would have had difficulty affecting the surface of each cage structure. Thus, the samples were firstly ground to a small size (0.1–1 µm) using air jet milling (AJM, 2-inch Sturtevant micronizer) at 90 PSI pressure, prior to the functionalization.

### 2.3. Plasma Functionalization by Stirring Plasma Treatment

**Figure 1:**
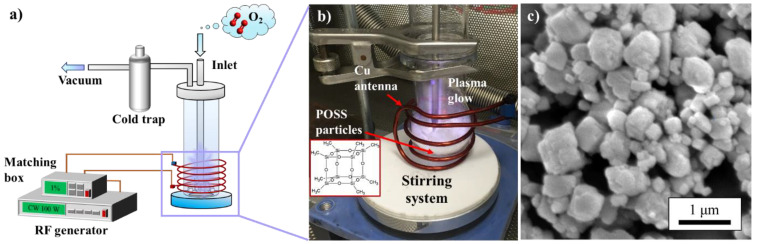
The octa-methyl POSS sub-micron particle with its chemical structure and the bespoke low-pressure stirring plasma system: (**a**) overview scheme for the plasma system installation; (**b**) a photo of the plasma glow for POSS particles in the reaction chamber; (**c**) SEM images of octa-methyl POSS sub-micron particles.

The low-pressure stirring plasma system was customized for the surface functionalization of powder samples as shown in Figure 1a,b. A Pyrex cylindrical chamber (DI: 40 mm, length: 400 mm) was connected to a round flask at one terminal of the chamber. The round flask works as the sample holder in the plasma treatment system. The stirring process was controlled by a magnetic stir bar and coupled stirrer in the round flask which achieved a continuous exposure of the sample surface to the plasma glow. A copper wire antenna (DI: 3.74 mm) surrounded the round flask and connected to the radio-frequency-type (RF) plasma generator (13.56 MHz, Kurt J. Lesker Co., Jefferson Hills, PA, USA) with a matching box in a Faraday cage.

An average 60 mg of the octa-methyl POSS was used for each treatment by the stirring plasma process. The working pressure was 8 × 10^−2^ mbar for the oxygen gas plasma functionalization as well as the Ar gas plasma pre-treatment. As can be seen in Table 1, plasma functionalization was investigated under different conditions, namely different plasma modes, RF powers, and processing durations. In addition, the pre-treatment was 3 min for all samples, and the duty cycle (DC) for pulsed plasma was 10% which was calculated from t_on_/(t_on_ + t_off_), in which t_on_ is 0.05 ms [24].

### 2.4. Characterizations

The morphology of the POSS particles was acquired using scanning electron microscopy (SEM, Jeol JSM 7800F) with a 5 kV accelerating voltage and 87 pA currents. The working distance for the SEM images was 12 mm. To avoid charging issues, a 3–4 nm sputtered gold coating was applied to the POSS samples before SEM.

The composition of the POSS was analyzed using a Fourier transform infrared spectrophotometer in the attenuated total reflectance mode (FTIR-ATR, Burker Vertex 70). The measured spectra were in the range of 600–4000 cm^−1^ with 128 scans and 4 cm^−1^ resolutions. The corresponding chemical structure of the POSS was defined using locations in the spectra after normalization at 1075–1135 cm^−1^, when the main Si-O-Si structure was assumed to be stable after processing. The assignment of FTIR band is summarized in Table 2.

The surface composition was quantitatively analyzed by X-ray photoelectron spectroscopy (XPS, K-Alpha Thermo Fisher Scientific, Waltham, MA, USA at RMIT University and Kratos AXIS Nova at Latrobe University). During the measurement, the chamber pressure was controlled to around 5 × 10^−9^ mbar with a scan spot size of 400 μm, and the flood gun was switched on to reduce the charging issue. Each high-resolution elemental peak spectrum was scanned 10 times with a voltage of 20 eV and analyzed by CasaXPS software (version 2.3.18) using the Gaussian–Lorentzian (G/L) model. In addition, the approximate number of carboxyl and hydroxyl groups was estimated from their fitted areas in peaks compared to the C-H peak in the high-resolution C1s spectra.

## 3. Results

### 3.1. Plasma Strategies for “Green” Functionalization on Octa-Methyl POSS

During a CW + P oxygen plasma process, the CW plasma generates reactive oxygen species from oxygen gas with high kinetic energy which constantly bombards the surface of the POSS [28,41,42]. The pulsed plasma provides milder kinetic energy from plasma owing to a frequently repeated duration of gas discharge on-time and off-time [24,34,37]. Neutral radicals remain owing to their longer lifetime compared to ions and electrons during the off-time in pulsed plasma, which suggests the formation of functional groups from those reactive radicals [43]. Thus, the combination of CW and P in oxygen plasma suggests the activation of POSS microstructures from the CW mode, and the continuous introduction of designable functional groups from the P mode [37]. With the assistance of stirring, oxygen gas in various plasma modes was applied to the octa-methyl POSS sub-micron powder to investigate the feasibility of the introduction of oxygen moieties. The SEM image confirms that POSS powder after grinding offered a crystalline particle from 100 nm to 1000 nm (Figure 1c). The morphology remained stable after plasma treatment, and the details of the SEM results can be found in our previous work [24].

**Figure 2:**
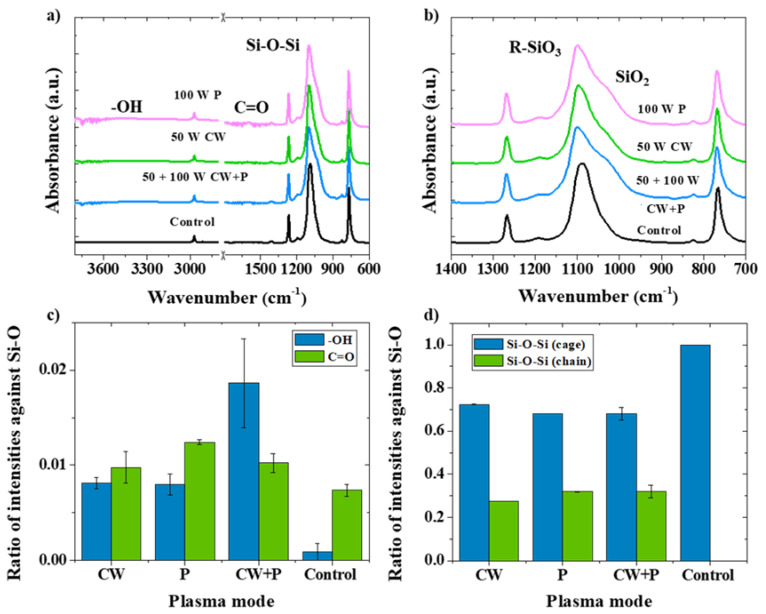
Chemical bonding structure analysis by FTIR for various modes with consistent applied RF energies for oxygen plasma of 550 W·min compared to the control POSS: (**a**) the full range of spectra, 600–4000 cm^−1^; (**b**) a small range of FTIR spectra, from 700 to 1400 cm^−1^; (**c**) intensity ratios for C=O and -OH as against Si-O; (**d**) intensity ratios for cage and opened-cage (chain) identities.

Figure 2a shows the FTIR spectra for POSS samples treated with oxygen gas plasma using three different plasma modes compared with the control sample. The influence on the core-cage Si-O band is found from the location 1030–1100 cm^−1^, which is slightly wiggly after the application of oxygen active species (Figure 2b). The C=O (carbonyl) and -OH (hydroxyl) are also found at 1610 and 3300 cm^−1^, respectively [27]. The H atoms on the methyl groups of the octa-methyl POSS microstructures were tuned by oxygen species which form OH radicals and bond with C atoms represented as hydroxyl groups [28]. In addition, the C=O band could also correspond to part of the carboxyl groups (O-C=O) [27]. Moreover, the effectiveness of the plasma modes in generating oxygen-rich functional groups can be related to the normalized band intensities in the FTIR spectra (Figure 2c). Specifically, ratios from normalized intensities for C=O and -OH against core-cage Si-O indicate the relatively high amount of them compared to the control POSS [31]. As a result, all three plasma modes used in the stirring of POSS sub-micron particles provided high ratios for C=O and -OH of more than 42.8% compared to the control particles. Furthermore, compared with CW and P modes, the CW + P plasma yielded an increase of 147% for -OH which confirms the benefits of using P plasma after CW plasma. The POSS cage structure can be defined in the range from 1030 to 1100 cm^−1^ (Figure 2b), which are assigned as cage identity (R-SiO_3_) and opened-cage identity (SiO_2_) [44]. As a major band for native octa-methyl POSS, 1080 cm^−1^ represents the cage feature, while the 1030 cm^−1^ shoulder indicates damaged cages [44,45]. Hence, as can be seen in Figure 2d, the intensity ratios can be summarized in terms of cage identity and opened cage (chain) identity. When the ratio of Si-O-Si (chain) becomes higher than that of Si-O-Si (cage), octa-methyl POSS particles may lose their integrity [45]. Therefore, kinetic energies applied from three oxygen plasma treatments only reaches the breakdown energy of C-H bonding without damaging the Si-O core structure. The successful functionalization with oxygen-rich functional groups is also indicated by the results. In addition, because the source of plasma is mainly based on the input gas, the use of other highly reactive chemicals is unnecessary.

### 3.2. The Introduction of Hydroxyl Groups (OH) in CW Plasma

The CW plasma offers a continuous bombardment of the octa-methyl POSS sub-micron particles by reactive oxygen species, which suggests both etching and generation of active sites as well as formation of functional groups [35]. Different RF powers for CW plasma, namely 50, 60, 80, and 100 W, were studied to investigate the influence from all kinds of reactive oxygen species on POSS microstructures.

**Figure 3:**
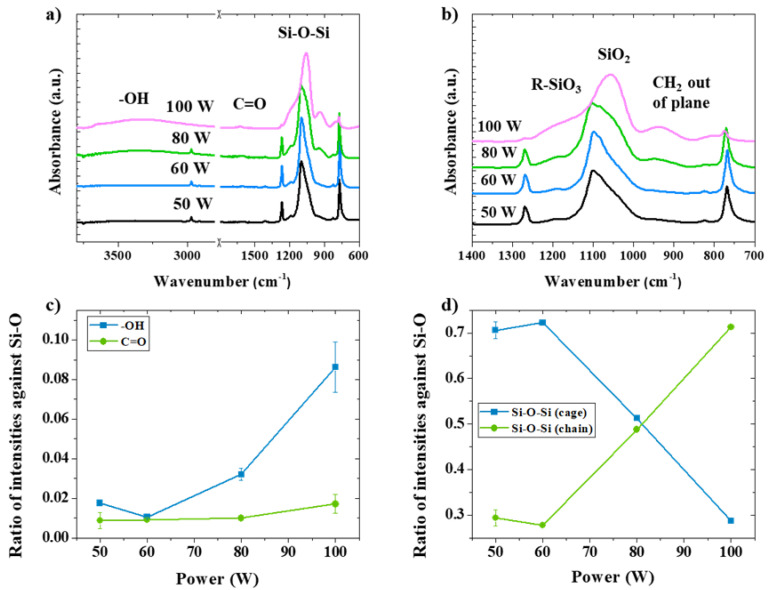
Chemical bonding structure analysis by FTIR for various RF powers in CW oxygen plasma with consistent processing durations: (**a**) the full range of spectra, 600–4000 cm^−1^; (**b**) a small range of FTIR spectra, from 700 to 1400 cm^−1^; (**c**) intensity ratios for C=O and -OH as against Si-O; (**d**) intensity ratios for cage and opened-cage (chain) identities.

The FTIR spectra of the octa-methyl POSS after different RF powers for CW plasma can be seen in Figure 3a,b. Oxygen-rich moieties are indicated in all treated POSS particles through the appearance of a bonding structure of C=O and -OH (Figure 3a). Moreover, intensity ratios of C=O/Si-O and -OH/Si-O from the FTIR spectra are estimated as 90% and 350% higher for RF power of 100 W in comparison with 50 W, respectively (Figure 3c). Both the appearance of CH_2_ out of plane (950 cm^−1^) and a decline of intensity of Si-CH_3_ (2700–2990 cm^−1^) are visible after plasma treated by 100 W of RF power input, implying the dissociation of CH_3_ sites. Therefore, a negative effect on the integrity of the POSS cage can be seen at high kinetic energy from RF power of 100 W, leading to Si-C bond dissociation and towards Si-O bond opening when RF power exceeds 60 W. To further understand the implications for the cage structure integrity at above 60 W of RF power, intensity ratios for cage and opened-cage identities are evaluated and presented in Figure 3d. The results confirm a shift from cage structure to opened-cage for plasma treated by above 60 W of RF power, as seen from the higher ratio for SiO_2_ followed by terminal methyl groups dissociations in comparison with pristine POSS.

**Figure 4:**
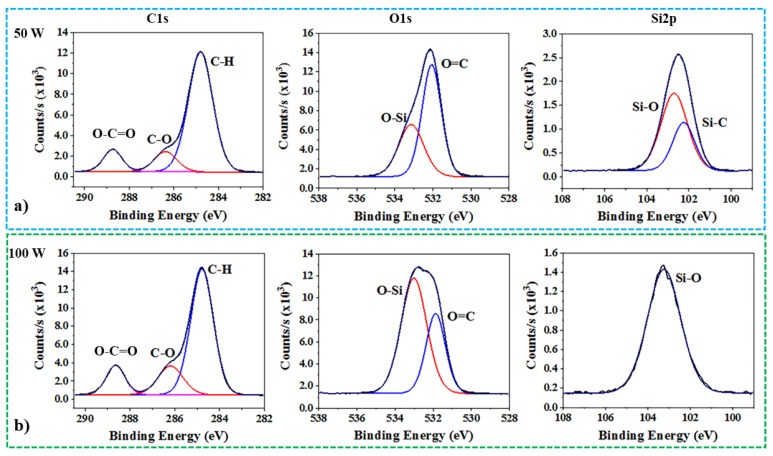
Surface chemical composition of oxygen CW plasma-treated POSS at various RF powers from XPS for C1s, O1s, and Si2p high-resolution spectra analysis: (**a**) for RF power of 50 W; (**b**) for RF power of 100 W.

The bonding energies of the octa-methyl POSS after oxygen plasma processing at 50 W and 100 W of RF powers in CW mode are examined by XPS analysis (Figure 4). The C-H bonding is determined at the main peak in C1s with 284.8 eV, while C-O (286.5 eV) and O-C=O (288.8 eV) are found after oxygen plasma treatments [27,28]. The O-C=O bonding that can be seen from the XPS analysis agrees with the carbonyl (C=O) signal from the FTIR [28]. Hence, the C=O signal is highly possibly a part of O-C=O that was introduced by the oxygen plasma. Since there is no bonding between carbon and oxygen in the pristine octa-methyl POSS, C-OH from hydroxyl is confirmed while O-C=O from carboxyl is also confirmed [45]. When the RF power for CW plasma was 100 W, the increased amount of hydroxyl and potential carboxyl were found through the change in XPS spectra, specifically from C-O and O-C=O peaks. Owing to the appearance of Si-C (102.1 eV) and Si-O (102.9 eV) peaks after 50 W of RF power for plasma from POSS, the nature of the cage is maintained. However, a strong peak for O-Si in O1s is fitted when the RF power is 100 W compared to 50 W, implying damage to the cage structure [22,24]. As can be seen in the Si2p spectra, a unique peak is indicated for Si-O (103.1 eV), which is from the opened cage structure of the POSS [45,46]. Hence, the continuous etching on the POSS cage by reactive oxygen species leads to damage from Si-O bonding. The increased number of oxygen-rich functional groups locate at the increased number of active sites from the opened corner of the cubic Si-O-Si structure. Therefore, oxygen CW plasma processing using an RF power of lower than 60 W achieves the preferential introduction of -OH radicals on octa-methyl POSS sub-micron particles without the loss of their core cage structure.

### 3.3. The Introduction of Carboxyl Groups (COOH) in Combined Mode Plasma

The combined plasma mode (CW + P) suggests a higher functionalization efficiency on nanofillers [24,37]. The additional pulsed plasma after CW plasma increased the number of long-living neutral reactive species able to form radicals between O atoms and etched atoms from the methyl groups of the POSS. Thus, using combined mode could increase the generation of hydroxyl (OH) and carboxyl (COOH) on octa-methyl POSS microstructures. An RF power of 50 W applied to CW plasma was used in order to maintain the cage structure and activate the surface of the octa-methyl POSS sub-micron particles.

**Figure 5:**
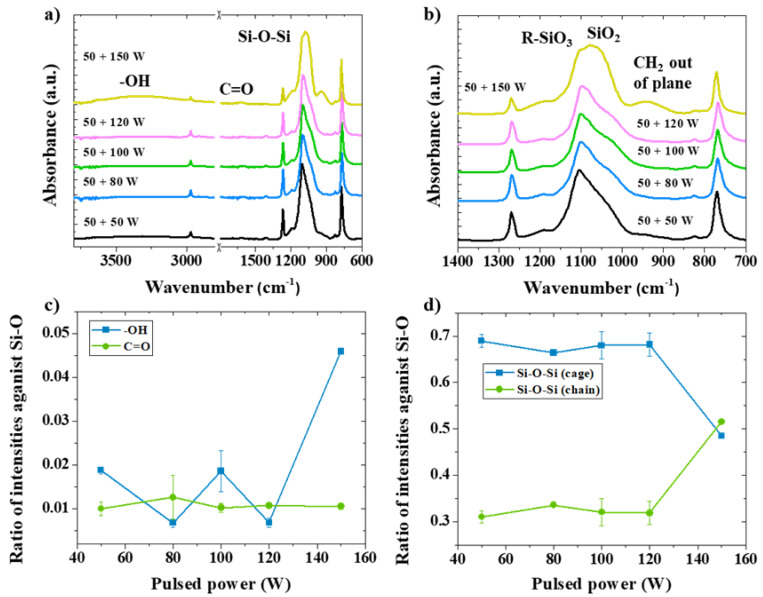
Chemical bonding structure analysis by FTIR for various RF powers in pulsed plasma mode after CW oxygen plasma (CW + P) with consistent conditions: (**a**) the full range of spectra, 600–4000 cm^−1^; (**b**) a small range of FTIR spectra, from 700 to 1400 cm^−1^; (**c**) intensity ratios for C=O and -OH as against Si-O; (**d**) intensity ratios for cage and opened-cage (chain) identities.

-OH and C=O signals are found in FTIR measurements from all treated POSS sub-micron particles owing to the oxygen species bombardment (Figure 5a,b). It can be seen from the intensity ratios against Si-O that C=O bonding is observed and shows a small variation from 50 to 150 W of RF power for pulsed plasma (Figure 5c). Meanwhile, the -OH content is increased by 4.5 times at 150 W of RF power for pulsed plasma in comparison with other RF powers. However, there is an offset of wavenumber in the FTIR spectra from 1080 to 1030 cm^−1^ for Si-O bonding after treatment at RF power of 150 W, which represents opening of the POSS cage structure [45]. Hence, for plasma-treated POSS in the combined mode at RF power of 150 W, the intensity ratio for cage identity decreases by 29.0% while the ratio for damaged cage identity increases at the same rate, as shown in Figure 5d. Owing to the increase in RF power for pulsed plasma, the applied kinetic energy was high which caused the dissociation of the Si-O-Si structure. Therefore, retention of cage integrity in octa-methyl POSS and a potentially high amount of -OH and C=O bonding are suggested from 50 W of RF power for CW mode and 100 W of RF power for pulsed mode.

**Figure 6:**
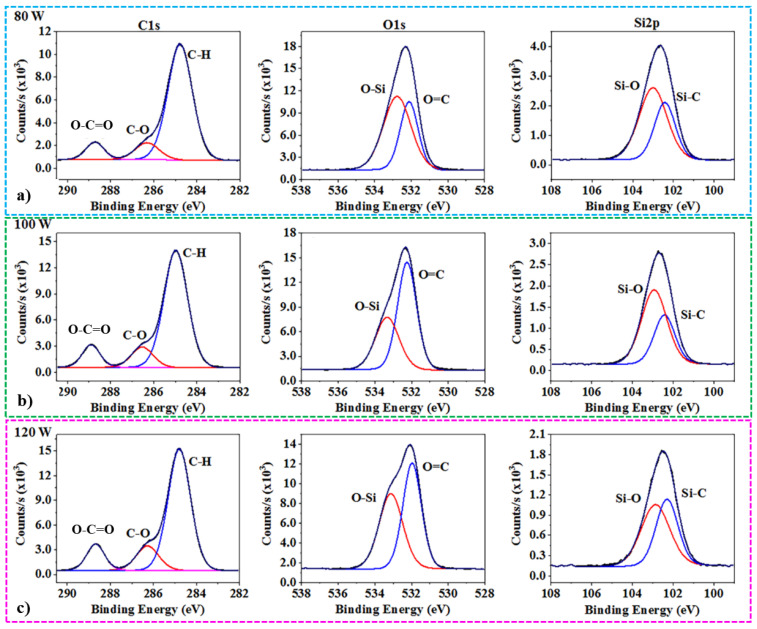
Surface chemical composition of oxygen CW + P plasma-treated POSS for various RF powers in pulsed mode from XPS for C1s, O1s, and Si2p high-resolution spectra analysis: (**a**) for pulsed RF power of 80 W; (**b**) for pulsed RF power of 100 W; (**c**) for pulsed RF power of 120 W.

Moreover, XPS measurements confirmed the chemical composition of the POSS after treatment with from 80 to 120 W of RF powers used in pulsed mode from the CW + P oxygen plasma functionalization (Figure 6). For all of the treatments, the proportions for hydroxyl (C-O) and carboxyl (O-C=O) peaks in the C1s spectra are presented. It can be seen that O=C peaks in the O1s spectra continuously increase as an increased number of carboxyl groups are grafted on to the POSS with the increased RF power for plasma [28]. In addition, the integrities of the POSS cages can be sustained through the same locations for Si-C and Si-O in the Si2p spectra [22,46]. The XPS analysis shows that the total Si composition is also decreased by oxygen plasma, which also indicates an increase in active sites for grafting carboxyl or hydroxyl groups onto the surface of the octa-methyl POSS sub-micron particles.

**Figure 7:**
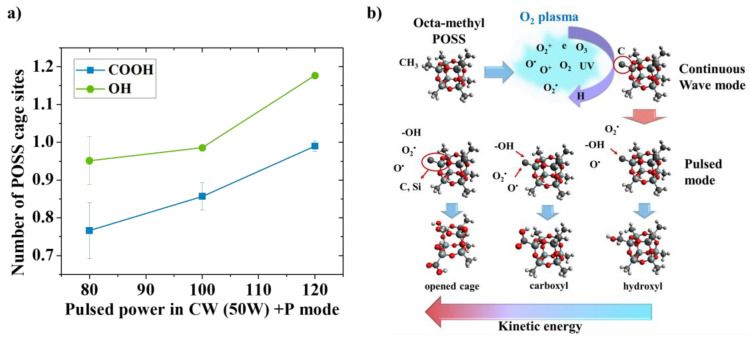
(**a**) Estimated number of sites of oxygen moieties grafted onto the POSS cage corners for various RF powers in pulsed mode from CW + P oxygen plasma processing; (**b**) potential mechanism of plasma functionalization by using oxygen gas on the POSS cage.

The number of carboxyl and hydroxyl groups was quantified by XPS measurements performed on the plasma-treated POSS sub-micron particles (Figure 7). The fitted peaks in the C1s spectra can be used to identify the relative numbers of different functional groups in the octa-methyl POSS. After CW plasma activation using 50 W of RF power is applied, a continuous increase in O-C=O and C-OH is observed when the RF power for pulsed plasma increases (Figure 7a). Application of 100 W of RF power to the pulsed plasma treatment in the combined mode suggests nearly one hydroxyl group per POSS microstructure compared to the pristine, while the carboxyl bonding is less than 1. Application of 120 W of RF power in the pulsed plasma treatment of the sample in the combined plasma mode suggests a 28.9% increase for the O-C=O peak—that is, one carboxyl group per POSS microstructure compared to the application of 80 W of RF power to the pulsed plasma treatment.

The potential mechanism for functionalization by oxygen gas plasma in CW + P mode is depicted in Figure 7b. The octa-methyl POSS sub-micron particles were firstly stirred prior to the gas discharge to optimize treatment homogeneity. The CW plasma offers a partial ionization of the oxygen gas into various reactive oxygen species, including atoms and molecules, ions, electrons, and radicals [47]. They contain relatively high kinetic energy that bombards the surface of particles resulting in the C-H of methyl groups from the octa-methyl POSS being compromised and reacting with the O species to form -OH radicals and further forming C=O bonding on POSS [28]. Owing to the highly reactive species in the CW plasma, the formation of functional groups and the etching of the surface occur at the same time [33]. Thus, there will be a limit to the number of functional groups grafting at a given applied energy before the Si-O-Si structure starts degrading. The subsequent P plasma of oxygen gas supplies relatively low kinetic energy and forms long-living neutral O species [34]. Owing to the active sites formed from the CW plasma, reactive radicals create an increased number of -OH and other oxygen-rich functional groups that can easily graft onto the POSS microstructures. With an increase in RF power for the pulsed plasma (> 100 W), the applied kinetic energy is increased, which results in more chance of forming O-C=O carboxyl groups [28]. In addition, when the RF power for pulsed plasma is further increased, the core cage structure of POSS is degraded because it reaches a sufficient amount of kinetic energy to dissociate the bonds [24]. In this study, it has been shown that the kinetic energy applied from pulsed plasma in the combined mode is important for the introduction of the hydroxyl to carboxyl groups on the octa-methyl POSS sub-micron particles. More importantly, this work has shown that the stirring plasma route can provide precise control of the density of the functional groups on octa-methyl POSS without lengthy wet-chemical modifications.

## 4. Conclusions

A low-pressure stirring plasma system was investigated as a strategy to functionalize octa-methyl POSS sub-micron particles with hydroxyl and carboxyl groups. By carefully controlling the energy input during the combined plasma mode, it has been demonstrated that one hydroxyl group or one carboxyl group can be grafted by plasma onto the corner of the POSS cage without loss of the cage structure integrity. The successful hydroxyl and carboxyl groups could be crosslinked and used as spacers for anti-fouling and permeation improvement in membrane materials. Further enhancement of the density of the functional group can be achieved through tuning of the treatment duration. This “green” plasma offers a much more environmentally friendly approach compared to other wet-chemistry approaches, and it could be applied in the next-generation fabrication of functional materials as part of a cost-effective pathway.

## Figures and Tables

**Table 1:** Experimental conditions for oxygen stirring plasma functionalization.

Plasma Treatments	CW Conditions		P Conditions	
	RF Power (W)	Duration (min)	RF Power (W)	Duration (min)
**Various plasma modes**	50	11	-	
	-		100	55
	50	5	100	30
**Various RF powers for CW plasma**	50	5	-	
	60	5	-	
	80	5	-	
	100	5	-	
**Various RF powers for P plasma in CW + P mode**	50	5	50	30
	50	5	80	30
	50	5	100	30
	50	5	120	30
	50	5	150	30

**Table 2:** The FTIR band locations and their corresponding chemical structure [24].

Assignment	Band Range (cm^−1^)
O-H (hydroxyl)	3200–3500
C-H	2700–2990
C=O (carbonyl or carboxyl)	1580–1650
C-H	1250–1280
Si-O	1075–1135
Si-C	720–860

## Data Availability

The data that support the findings of this study are available from the corresponding authors upon reasonable request.
